# Impact of leaving periodontal disease untreated on healthcare expenditures: A retrospective cohort study

**DOI:** 10.1002/jper.70136

**Published:** 2026-04-19

**Authors:** Anna Kinugawa, Kenji Takeuchi, Yudai Tamada, Taro Kusama, Futoshi Oda, Megumi Maeda, Hiroe Ohyama, Ken Osaka, Haruhisa Fukuda

**Affiliations:** ^1^ Department of International and Community Oral Health Tohoku University Graduate School of Dentistry Miyagi Japan; ^2^ Division of Statistics and Data Science Liaison Center for Innovative Dentistry Tohoku University Graduate School of Dentistry Miyagi Japan; ^3^ Department of Health Care Administration and Management Kyushu University Graduate School of Medical Sciences Fukuoka Japan; ^4^ Department of Restorative Dentistry and Biomaterials Sciences Harvard School of Dental Medicine Boston Massachusetts USA

**Keywords:** dental public health, epidemiology, health care costs, health service research, periodontal diseases, preventive dentistry

## Abstract

**Background:**

This study aimed to investigate whether healthcare expenditures (HCEs) differed depending on whether the patients left periodontal disease (PD) untreated, despite the need for treatment.

**Methods:**

This study used public PD screening data from a municipality in Japan to identify adults aged ≥ 40 years who were found to require treatment of PD by dentists at PD screening. The presence or absence of periodontal treatment was determined by a dental visit within 180 days after the date of PD screening based on medical claims data. Annual HCE were calculated from cumulative expenditures over 2 years from the date of the presence or absence of periodontal treatment. A generalized linear model with a gamma distribution and log link function was used to calculate the relative cost ratios (RCRs) and 95% confidence intervals (95% CI), and a two‐part model was used to predict annual HCEs and the difference in predicted HCEs based on dental treatment.

**Results:**

Among 652 people (mean age: 62.6 years [1 SD = 9.0], 65.5% women), 9.0% were untreated. After adjusting for the covariates, the RCR for medical, pharmaceutical, and dental costs in the untreated group compared with the treated group were 1.56 (95% CI: 1.02–2.38), 0.95 (95% CI: 0.53–1.70), and 0.14 (95% CI: 0.10–0.19), respectively. The differences in predicted HCEs were $593.5 (95% CI: –280.6, 1467.6) higher for medical, $79.9 (95% CI: –363.6, 523.4) higher for pharmaceutical, and $323.9 (95% CI: –397.3, –250.6) lower for dental.

**Conclusion:**

Leaving PD untreated was associated with increased HCEs, particularly medical expenditures.

**Plain language summary:**

This study looked at whether people who were told by a dentist that they needed care for periodontal disease actually received treatment, and how this affected their healthcare costs. The research followed more than 600 adults in Japan for 2 years, comparing the cumulative healthcare costs of those who received dental care with those who did not. The results showed that people who did not treat their periodontal disease ended up spending more on medical care, even though they spent less on dental care. On average, untreated individuals had medical expenses that were around $600 higher than those who received treatment. This suggests that avoiding dental care for periodontal disease might lead to more serious health problems down the line, which can increase overall healthcare costs. Although skipping dental visits may seem like a way to save money, it could actually lead to higher medical bills in the future. These findings highlight the importance of early dental treatment not just for oral health, but also for managing overall healthcare costs and preventing other health complications.

## 1 INTRODUCTION

Out of more than 300 diseases and conditions, oral diseases, represented by untreated caries and severe periodontitis, are the most prevalent condition in the world, with huge impacts on other health conditions and the economy.^1^ A recent report estimated that the total worldwide economic impact of oral diseases was USD 710 billion, with USD 387 billion due to direct costs.^2^ However, oral diseases are considered separate from other health conditions and their impact on the economy is often neglected.^3^, ^4^

The treatment of oral diseases may lead to reduced healthcare expenditure. Previous studies have reported that patients with diabetes who received periodontal treatment had lower healthcare expenditures than those who did not.^5^, ^6^, ^7^ However, because the classification of the treated and untreated groups in these studies was based solely on claims data, the untreated group included healthy people who did not require treatment; in other words, people who did not have periodontal disease. This may have overestimated the impact of periodontal treatment on healthcare expenditures. Thus, there is insufficient evidence regarding the effect of periodontal treatment on healthcare expenditures for patients requiring treatment.

In this study, by targeting a population diagnosed as requiring treatment of periodontal disease at a periodontal disease (PD) screening, we aimed to investigate whether healthcare expenditure differed depending on whether they received the treatment after the screening. The hypothesis was put forward that postponing dental treatment, that is, leaving periodontal disease untreated, leads to an increase in medical expenses.

This study was approved by the Kyushu University Institutional Review Board for Clinical Research (approval No. 2214‐09) and the Ethics Committee of Tohoku University Graduate School of Dentistry (approval No. 37148). Participant consent was collected via an opt‐out process, and the requirement for individual informed consent was waived because only anonymized data were used. A Clinical Study Registration Number was not applicable to this study because of its retrospective observational design.

## 2 METHODS

### 2.1 Setting and participants

This retrospective cohort study used data from the Longevity Improvement Fair Evidence (LIFE) Study,^8^ a longitudinal community‐based database project that collected administrative healthcare (dental, medical, and pharmacy) claims data and public dental health checkups data, such as PD screening data,^9^ in collaboration with municipal governments. In the LIFE Study, a unique identifier was assigned to each participant by the data managers, and each data point was linked at the individual level using a pseudonymized personal identifier. In Japan, people are mandated to enroll in a health insurance covering dental services under the Japanese health insurance system. We used the data of enrollees of the National Health Insurance (NHI), which is a public insurance scheme primary maintained by municipal governments for the self‐employed or not employed, including part‐time workers and retired individuals, and their dependents aged ≤ 75 years. The Japanese health insurance system is described in detail elsewhere.^10^ Periodontal disease screening was conducted under the Health Promotion Act for middle‐aged populations, regardless of their health insurance type. During screening, dentists assessed the teeth, gingiva, plaque, and dental prosthesis of the participants. After checking the oral conditions, participants were categorized into one of three groups based on their oral condition: (i) “further treatment recommended” which included individuals with a periodontal probing depth (PPD) of 4 mm or greater, or those with decayed or missing teeth; (ii) “oral hygiene instruction on site” which consisted of individuals who exhibited gingival bleeding or poor plaque control; and (iii) “no issues identified” which included those without oral health problems.

In this study, we used the healthcare claims data from June 2018 to April 2022 and PD screening data collected from a municipality in the fiscal year 2019 (offered from June to November 2019). In the municipality, the resident population is approximately 500,000–1,000,000 people, and individual PD screening is offered free of charge at designated dental clinics once every 5 years. The inclusion criteria were as follows: (i) those who underwent PD screening and received the result of “further treatment recommended” based on their PPD, and (ii) those aged ≥ 40 years. Those who withdrew from the NHI during the study period and had missing smoking status were excluded from the analysis. We also excluded those with extremely high healthcare expenditures (with a log‐transformed value exceeding 14.3 [USD 89021.1 to USD 132229.1], defined based on the distribution of healthcare expenditures) (Figure S1 in the online *Journal of Periodontology*). This is because these high healthcare expenditures were not directly related to the presence or absence of dental treatments.

**FIGURE 1:**
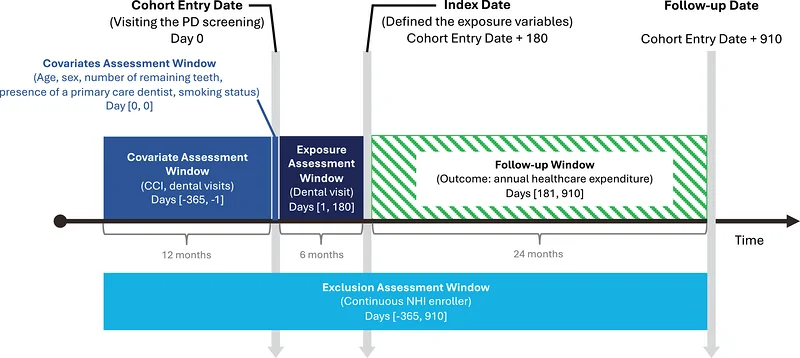
Graphical depiction of study design. CCI, Charlson Comorbidity Index; NHI, national health insurance; PD, periodontal disease.

Figure 1 presents the design diagram^11^ of this study. The cohort entry date was defined as the date of PD screening. The index date was set at 180 days after the cohort entry date to define the exposure variable, based on a previous study.^12^ The outcome variable was followed up for 2 years (730 days) after the index date (910 days after the cohort entry date as the follow‐up date).

### 2.2 Outcome variable

Healthcare expenditures for medical, dental, and pharmaceutical care during the follow‐up period were used as the outcome variables. Overall healthcare expenditure was defined as the sum of medical, dental, and pharmaceutical healthcare expenditures. Each healthcare expenditure was calculated as the sum of each claim for a 2‐year follow‐up period. Each annual healthcare expenditure was calculated by dividing the healthcare expenditures by two (follow‐up period). Participants who did not report claims data were considered to have healthcare expenditures of USD 0.

### 2.3 Exposure variable

Adherence to the treatment recommendations after PD screening was used as the exposure variable. Those who visited the dentist within 180 days after PD screening were allocated to the treated group, and those who had delayed or forgone were allocated to the untreated group. Dental visits were determined on the basis of the occurrence of dental claims. In Japan, initial‐visit patients with periodontal disease generally receive periodontal probing depth check, scaling, and root planing as part of routine care. Although all participants were identified as “further treatment recommended” based on their PPD at PD screening, some individuals also had other dental problems, such as dental caries and denture‐related problems, requiring treatment. In such cases, the management of non‐periodontal conditions may precede the initiation of periodontal therapy in routine clinical practice. Because the dental claims data do not provide information on the overall treatment plan at the initial visit, we used the occurrence of a dental visit following PD screening as a proxy for having received periodontal treatment.

### 2.4 Covariates

The covariates included age, sex, number of remaining teeth, dental visits in the past year, the presence of a primary care dentist, smoking status, and updated Charlson Comorbidity Index (CCI) score.^13^ Age, sex, number of remaining teeth, the presence of a primary care dentist, and smoking status were recorded during the PD screening. Smoking status was assessed by self‐reported questionnaires with “yes” or “no” responses. Dental visits in the past year were identified by the issuance of dental claims within 1 year before PD screening. The CCI score was assessed using claims data for 1 year before PD screening. CCI score was categorized as 0, 1, or ≥ 2.

### 2.5 Statistical analysis

A generalized linear model (GLM) with gamma distribution and a log link function was used to estimate the relative cost ratios (RCRs) and 95% confidence intervals (CIs) for subsequent healthcare expenditures by individuals with and without dental treatment. Additionally, a two‐part model was used to estimate the annual healthcare expenditures of the treated and untreated groups and their differences based on the parametric‐g formula.^14^ The two‐part model consisted of a logistic regression model for the first part, modeling the probability of incurring any healthcare expenditure, and a gamma model with an identity link function for the second part, modeling the level of healthcare expenditure among those with positive expenditures. To estimate the overall marginal effect of exposure on healthcare expenditure, we used the *margins* command to derive the combined expected values from the two‐part model. Average marginal effects and 95% CIs were calculated. Although possible confounders were included in the analysis, there is a possibility that unmeasured confounding factors affected the results. Therefore, we calculated the E‐values of the RCR for each expenditure to identify the strength of the unmeasured covariates that affected the estimates.^15^ As a sensitivity analysis, we conducted inverse probability weighting (IPW) analysis to balance baseline participant characteristics between the treated and untreated groups.^16^ Propensity scores were estimated using a logistic regression model including baseline covariates. The inverse probability of treatment weights was then computed based on the estimated propensity scores. To improve the stability of the estimates, stabilized weights were applied, and extreme weights were trimmed at the 1st and 99th percentiles.^17^ The weighted GLM and weighted two‐part models were then re‐estimated using these weights. All data were analyzed using STATA (version 17.0; Stata Corp., College Station, TX, USA). The level of significance was set at alpha = 0.05. This study conformed to STROBE guidelines.

## 3 RESULTS

**FIGURE 2:**
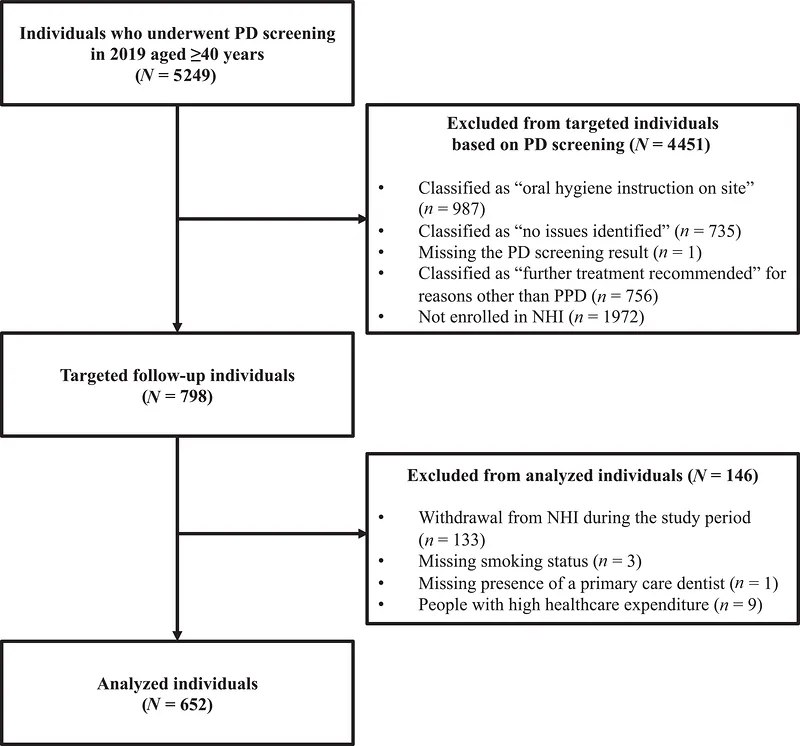
Flowchart of study participant inclusion. NHI, national health insurance; PD, periodontal disease; PPD, periodontal probing depth.

Figure 2 shows a flowchart of participant inclusion. Finally, 652 participants (mean age: 62.6 (SD = 9.0) years; 65.5% women) were included in the analysis. Of the participants, 91.0% were classified into the treated group and 9.0% into the untreated group. Table 1 presents the characteristics of the study participants. The untreated group was more likely to be younger (aged 40–59 years), men, and to have had no dental visits in the past year. The mean annual healthcare expenditures were USD 1883.8 (SD = 3092.3) for medical, USD 790.0 (SD = 1501.1) for pharmaceutical, USD 410.6 (SD = 406.1) for dental, and USD 3084.4 (SD = 3730.8) for overall (using an exchange rate of 1 USD = 100 JPY).

**TABLE 1:** Characteristics of study participants (*n* = 652).

		Dental treatment		
	Total *n* = 652	Treated *n* = 593	Untreated *n* = 59	*p* value^a^
Age, *n* (%)				
40–49	50 (7.7)	42 (84.0)	8 (16.0)	0.081
50–59	124 (19.0)	109 (87.9)	15 (12.1)	
60–69	184 (28.2)	167 (90.8)	17 (9.2)	
≥70	294 (45.1)	275 (93.5)	19 (6.5)	
Sex, *n* (%)				0.057
Males	225 (34.5)	198 (88.0)	27 (12.0)	
Females	427 (65.5)	395 (92.5)	32 (7.5)	
Number of remaining teeth, *n* (%)				0.662
0–19 teeth	78 (12.0)	73 (93.6)	5 (6.4)	
20–27 teeth	371 (56.9)	337 (90.8)	34 (9.2)	
≥28 teeth	203 (31.1)	183 (90.1)	20 (9.9)	
Dental visit in the past year, *n* (%)				<0.001
Yes	192 (29.4)	151 (78.6)	41 (21.4)	
No	460 (70.6)	442 (96.1)	18 (3.9)	
Primary care dentist, *n* (%)				0.393
Yes	565 (86.7)	516 (91.3)	49 (8.7)	
No	87 (13.3)	77 (88.5)	10 (11.5)	
Smoking status, *n* (%)				0.931
Non‐smoker	550 (84.4)	500 (90.9)	50 (9.1)	
Smoker	102 (15.6)	93 (91.2)	9 (8.8)	
CCI score, *n* (%)				0.215
0	370 (56.7)	333 (90.0)	37 (10.0)	
1	98 (15.0)	87 (88.8)	11 (11.2)	
≥2	184 (28.2)	173 (94.0)	11 (6.0)	
Annual healthcare expenditures (USD), mean (SD)				
Medical healthcare expenditure	1883.8 (3092.3)	1866.4 (2990.6)	2058.3 (4001.0)	0.155
Pharmaceutical healthcare expenditure	790.0 (1501.1)	810.3 (1553.4)	585.9 (783.3)	0.145
Dental healthcare expenditure	410.6 (406.1)	445.5 (407.1)	60.2 (150.4)	<0.001
Overall healthcare expenditure	3084.4 (3730.8)	3122.2 (3677.7)	2704.4 (4243.4)	0.003

Abbreviations: CCI, Charlson Comorbidity Index; SD, standard deviation; USD, United States dollar.

^a^ *p* values were calculated using Pearson's *X* ^2^ test for age, sex, number of remaining teeth, dental visit in the past year, presence of a primary care dentist, smoking status, and CCI score, and Wilcoxon rank‐sum test for annual healthcare expenditures.

Table 2 shows the association between the dental treatment status and annual healthcare expenditure. The untreated group had a higher point estimate for medical expenditure (RCR: 1.56, 95% CI: 1.02–2.38) than that of the treated group. There was no difference in pharmaceutical expenditures (RCR: 0.95, 95% CI: 0.53–1.70) nor in overall healthcare expenditures (RCR: 1.09, 95% CI: 0.77–1.54) between the groups. Only the relative cost of dental healthcare expenditure was lower in the untreated group than in the treated group (RCR: 0.14, 95% CI: 0.10–0.19).

**TABLE 2:** Relative cost ratio of annual healthcare expenditure by treated or untreated dental treatment (*n* = 652).

	Model 1	Model 2
	RCR (95% CI)	RCR (95% CI)
Medical healthcare expenditure		
Treated	1.00 (Ref.)	1.00 (Ref.)
Untreated	1.10 (0.71–1.71)	1.56 (1.02–2.38)^*^
Pharmaceutical healthcare expenditure		
Treated	1.00 (Ref.)	1.00 (Ref.)
Untreated	0.72 (0.44–1.19)	0.95 (0.53–1.70)
Dental healthcare expenditure		
Treated	1.00 (Ref.)	1.00 (Ref.)
Untreated	0.14 (0.10–0.18)^**^	0.14 (0.10–0.19)^**^
Overall healthcare expenditure		
Treated	1.00 (Ref.)	1.00 (Ref.)
Untreated	0.87 (0.63–1.20)	1.09 (0.77–1.54)

Model 1 was crude model.

Model 2 was adjusted for age, sex, number of remaining teeth, dental visit in the past year, presence of a primary care dentist, smoking status, and CCI score.

Abbreviations: CCI, Charlson Comorbidity Index; CI, confidence interval; RCR, relative cost ratio; Ref, reference.

* *p* < 0.05.

** *p* < 0.001.

The results of the average marginal effects of dental treatment on annual healthcare expenditures (Table 3) showed that the untreated group was associated with USD 593.5 (95% CI: –280.6, 1467.6) higher for medical healthcare expenditures, USD 79.9 (95% CI: –363.6, 523.4) higher for pharmaceutical healthcare expenditures, and USD 148.1 (95% CI: –980.5, 1276.8) for overall healthcare expenditures relative to the treated group. Dental healthcare expenditures in the untreated group were significantly lower USD 323.9 (95% CI: –397.3, –250.6) than in the treated group. The results of the two‐part model for the full regression estimates are presented in Table S1 in the online *Journal of Periodontology*.

**TABLE 3:** Average marginal effect of dental treatment on annual healthcare expenditure estimated by two‐part model (*n* = 652).

	Predicted annual healthcare expenditures (USD)	95% CI	Marginal effects (USD)	95% CI
Medical healthcare expenditure				
Treated	1817.7	(1608.5, 2026.8)	Ref.	
Untreated	2411.2	(1542.2, 3280.2)	593.5	(–280.6, 1467.6)
Pharmaceutical healthcare expenditure				
Treated	794.5	(680.4, 908.6)	Ref.	
Untreated	874.4	(454.4, 1294.4)	79.9	(–363.6, 523.4)
Dental healthcare expenditure				
Treated	438.4	(406.7, 470.2)	Ref.	
Untreated	114.5	(49.9, 179.1)	−323.9^*^	(–397.3, –250.6)
Overall healthcare expenditure				
Treated	2473.6	(2165.7, 2781.5)	Ref.	
Untreated	2621.8	(1533.4, 3710.1)	148.1	(–980.5, 1276.8)

*Note*: Marginal effects were derived from a two‐part model. Estimates adjusted for age, sex, number of remaining teeth, dental visit in the past year, presence of a primary care dentist, smoking status, and CCI score.

Abbreviations: CCI, Charlson Comorbidity Index; CI, confidence interval; Ref, reference; USD, United States dollar.

* *p* < 0.001.

The point estimates of the calculated E‐values of the adjusted RCR for medical, pharmaceutical, dental, and overall healthcare expenditures were 2.50, 1.29, 13.77, and 1.40, respectively (Table S2 in the online *Journal of Periodontology*).

In sensitivity analyses using IPW to balance baseline characteristics between groups, the trend of the estimates was consistent with the main analysis (Table S3 in the online *Journal of Periodontology*).

## 4 DISCUSSION

This retrospective cohort study examined the differences in subsequent healthcare expenditures among individuals who required dental treatment during PD screening, based on whether they received treatment after screening. This study found that of those diagnosed as requiring treatment during PD screening, approximately 10% did not receive treatment and left their periodontal disease untreated. These individuals tended to have higher overall healthcare expenditures. In terms of healthcare expenditure components, the untreated group had significantly higher medical healthcare expenditures and significantly lower dental healthcare expenditures than the treated group, with no significant difference in pharmaceutical healthcare expenditures.

Our findings are consistent with those of previous cohort studies that found that people who did not receive dental treatment had higher medical healthcare expenditures than those who did.^5^, ^6^, ^7^ However, these studies simply compared the treated and untreated groups without considering whether the subjects had periodontal disease. Our findings enhance existing knowledge by providing new evidence on the impact of healthcare expenditures on those with periodontal disease who receive and do not receive treatment. In addition, a recent study that investigated the difference in medical costs between those with and without periodontal treatment, targeting only those with comorbid diseases, reported that medical costs in the periodontal treatment group tended to be lower than those in the untreated group among patients with diabetes.^18^ Since diabetes was the most common comorbid disease among our study participants, it can be interpreted that the tendency for medical costs to be lower in the treatment group was the same in both studies.

One possible pathway to explain the observed association between leaving periodontal disease untreated and increased medical healthcare expenditures could be comorbid chronic diseases. A previous review reported that people with periodontal disease are more likely to have chronic disease comorbidities.^19^ Leaving periodontal disease untreated may contribute to systemic inflammation, which may present or exacerbate systemic diseases such as diabetes, which incur high medical expenditures.^19^, ^20^, ^21^ In support of this, we recently reported that people with both periodontal disease and diabetes had higher healthcare expenditures than those without periodontal disease and those with only one disease.^22^ To further explore which components of medical care contributed to the observed differences in medical healthcare expenditures between the periodontal treatment and untreated groups, we examined the top five most frequently billed medical service procedure codes (Table S4 in the online *Journal of Periodontology*). Individuals in the untreated group had approximately twice the frequency of management fee for specific diseases, including those for chronic diseases such as diabetes mellitus, compared with the treated group. Because this billing code is typically applied to conditions requiring ongoing clinical management, our findings suggest that leaving periodontal disease untreated may be associated with greater clinical management needs for systemic diseases, thereby contributing to increased medical healthcare expenditures.

Our findings suggest that initiating dental treatment without delay in patients with periodontal disease who require treatment may lead to a reduction in healthcare expenditures. If the findings of this study are to be used from the perspective of public health, local governments that carry out PD screening should not only recommend that people who need dental treatment visit the dentist but also check whether they have visited the dentist after the screening by looking at their claims data. Local governments should continue to provide support until patients begin receiving dental treatment. In addition, in clinical settings, it is important for dental professionals to make efforts to reduce the possibility of treatment discontinuation by explaining to patients that leaving dental diseases untreated may lead to an increase in future healthcare expenditure.

This study has several limitations. First, the claims data did not include information on potential factors that could influence the participants’ patterns of dental visits, such as income and motivation to receive treatment. To evaluate the potential impact of unmeasured confounders, we calculated the E‐values of the RCRs for each expenditure (Table S2). The obtained E‐values indicated that, for medical healthcare expenditures, there are a few possible factors that explain the strong association between unmeasured confounders and both exposure and outcome. In contrast, the association with dental healthcare expenditures appear relatively robust. Second, we used a dental visit identified by dental claims occurrence as a proxy for receiving periodontal treatment. As a result, some dental visits may have been related to conditions other than periodontal disease, such as dental caries or trauma, leading to potential misclassification. However, to assess the validity of this proxy, we applied a previously established claims‐based definition of periodontal care^23^ and confirmed that more than 90% of individuals who visited a dentist following PD screening received periodontal‐related treatment. This finding supports the use of post‐screening dental visits as a reasonable indicator of periodontal treatment receipt in this study, although some degree of non‐differential misclassification cannot be completely excluded. Third, the maximum follow‐up period was limited to 2 years, which may be insufficient to fully capture the long‐term economic impact of periodontal treatment, particularly for chronic systemic diseases with long latency periods. In addition, because healthcare expenditures were calculated from aggregated monthly medical, dental, and pharmaceutical claims data, disease‐specific healthcare expenditures could not be reliably disaggregated. Consequently, longer‐term studies with more detailed, disease‐specific expenditure attribution are warranted to further elucidate the mechanisms underlying the observed associations. Fourth, our participants were limited to NHI enrollers who underwent PD screening in a single municipality. The consultation rate for PD screening was lower than other checkups (e.g., specific health check‐ups and cancer screening) and differed markedly across prefectures (0.34%–13.33%).^24^ Therefore, the generalizability and representativeness of our findings should be interpreted with caution.

## 5 CONCLUSION

In this study, almost all participants who required treatment visited a dentist after undergoing PD screening. Leaving periodontal disease untreated was associated with increased medical healthcare expenditure. Encouraging people who require dental treatment to visit dental clinics and initiate dental treatment could reduce healthcare expenditures.

## AUTHOR CONTRIBUTIONS

**Anna Kinugawa**: Conceptualization, formal analysis, methodology, writing — original draft, writing — review & editing. **Kenji Takeuchi**: Conceptualization, funding acquisition, methodology, project administration, supervision, writing — original draft, writing — review & editing. **Yudai Tamada**: Conceptualization, methodology, writing — review & editing. **Taro Kusama**: Conceptualization, methodology, writing — review & editing. **Futoshi Oda**: Data curation, investigation, writing — review & editing. **Megumi Maeda**: Data curation, investigation, writing — review & editing. **Hiroe Ohyama**: Conceptualization, methodology, writing — review & editing. **Ken Osaka**: Conceptualization, methodology, writing — review & editing, supervision, project administration, funding acquisition. **Haruhisa Fukuda**: Data curation, investigation, writing — review & editing, supervision, project administration, funding acquisition.

## CONFLICT OF INTEREST STATEMENT

The authors declare no potential conflicts of interest with respect to authorship and/or publication of this article.

## Supporting information



Supporting information

Supporting information

Supporting information

Supporting information

Supporting information

## Data Availability

All datasets used in this study are not publicly available due to ethical or legal restrictions. For inquiries regarding the datasets used in this study, please contact Dr. Haruhisa Fukuda, Principal Investigator of the LIFE Study, with reasonable requests.
