# Supplementary material for: Impact of leaving periodontal disease untreated on healthcare expenditures: A retrospective cohort study
Source: J Periodontol. 2026 Apr 19;97(9):2003–12. doi: 10.1002/jper.70136 (PMC13568914; doi:10.1002/jper.70136)
Supplement: Supplementary file 1 [file JPER-97-2003-s001.pdf]

Supplementary Table 2. Robustness to unmeasured confounding (E-values) on the association between dental treatment and subsequent annual healthcare expenditures (n = 652).

	Adjusted model†	
	**E-value for point estimate**	**E-value for confidence limit**
**Medical healthcare expenditure**		
Treated	(Ref.)	
Untreated	2.50	1.16
**Pharmaceutical healthcare expenditure**		
Treated	(Ref.)	
Untreated	1.29	1.00
**Dental healthcare expenditure**		
Treated	(Ref.)	
Untreated	13.77	10.00
**Overall healthcare expenditure**		
Treated	(Ref.)	
Untreated	1.40	1.00

†Adjusted for age, sex, number of remaining teeth, dental visit in the past year, presence of a primary care dentist, smoking status, and CCI score.
Abbreviation: Ref, reference; CCI, Charlson Comorbidity Index.
