# Supplementary material for: Impact of leaving periodontal disease untreated on healthcare expenditures: A retrospective cohort study
Source: J Periodontol. 2026 Apr 19;97(9):2003–12. doi: 10.1002/jper.70136 (PMC13568914; doi:10.1002/jper.70136)
Supplement: Supplementary file 2 [file JPER-97-2003-s002.pdf]

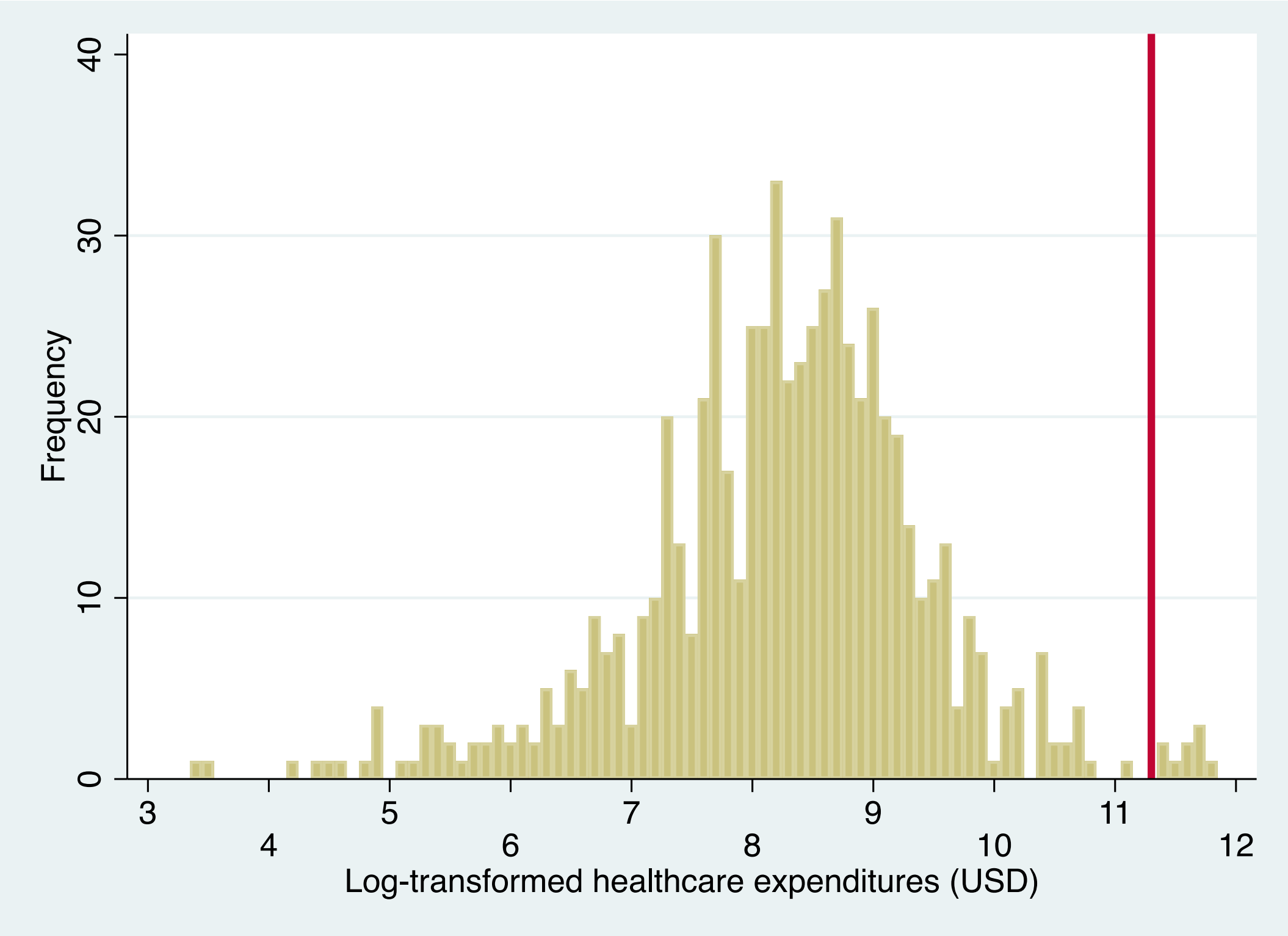


**Figure S1.** Distribution of log-transformed 2-year healthcare expenditures (n = 652).
Abbreviation: USD, United States dollar.
