# Supplementary material for: Impact of leaving periodontal disease untreated on healthcare expenditures: A retrospective cohort study
Source: J Periodontol. 2026 Apr 19;97(9):2003–12. doi: 10.1002/jper.70136 (PMC13568914; doi:10.1002/jper.70136)
Supplement: Supplementary file 3 [file JPER-97-2003-s003.pdf]

Supplementary Table 1. Results of two-part model for annual healthcare expenditure (n = 652).

	First-part		Second-part	
	**OR**	**95%CI**	**Coefficient**	**95%CI**
**Medical healthcare expenditures**				
Treated (Ref. Untreated)	0.04	-0.93, 1.00	633.4	-297.7, 1,564.5
**Pharmaceutical healthcare expenditures**				
Treated (Ref. Untreated)	-0.22	-0.95, 0.51	115.0	-398.7, 628.6
**Dental healthcare expenditures**				
Treated (Ref. Untreated)	-2.93***	-3.68, -2.17	-215.3**	-345.9, -84.7
**Overall healthcare expenditures**				
Treated (Ref. Untreated)	-1.01	-2.26, 0.25	234.3	-967.4, 1,436.0

*p < 0.05; **p < 0.01; ***p < 0.001

Note: Odds ratios are presented for the logit model; coefficients are shown for the GLM model.

Abbreviation: OR, Odds ratio; CI, confidence intervals; Ref, reference; GLM, Generalized linear model.
