# Supplementary material for: Impact of leaving periodontal disease untreated on healthcare expenditures: A retrospective cohort study
Source: J Periodontol. 2026 Apr 19;97(9):2003–12. doi: 10.1002/jper.70136 (PMC13568914; doi:10.1002/jper.70136)
Supplement: Supplementary file 4 [file JPER-97-2003-s004.pdf]

Supplementary Table 3. Results of sensitivity analyses using Inverse Probability Weighting (IPW) (n = 606*).

* Extreme weights at the 1st and 99th percentiles were observed in 46 individuals.

(a) Relative cost ratio of annual healthcare expenditures by treated or untreated dental treatment.

	**Model 1**<br>**RCR (95%CI)**	**Model 2**<br>**RCR (95%CI)**
**Medical healthcare expenditure**		
Treated	1.00 (Ref.)	1.00 (Ref.)
Untreated	1.22 (0.66–2.26)	1.80 (1.01–3.19)*
**Pharmaceutical healthcare expenditure**		
Treated	1.00 (Ref.)	1.00 (Ref.)
Untreated	0.71 (0.40–1.24)	0.92 (0.49–1.71)
**Dental healthcare expenditure**		
Treated	1.00 (Ref.)	1.00 (Ref.)
Untreated	0.28 (0.14–0.58)**	0.26 (0.14–0.50)***
**Overall healthcare expenditure**		
Treated	1.00 (Ref.)	1.00 (Ref.)
Untreated	0.96 (0.57–1.59)	1.15 (0.72–1.83)

*$p < 0.05$; **$p < 0.01$; ***$p < 0.001$
Model 1 was crude model.
Model 2 was adjusted for age, sex, number of remaining teeth, dental visit in the past year, presence of a primary care dentist, smoking status, and CCI score.
Abbreviation: RCR, relative cost ratio; CI, confidence intervals; Ref, reference; CCI, Charlson Comorbidity Index.

(b) Average marginal effect of dental treatment on annual healthcare expenditures estimated by two-part model.

	**Predicted Annual Healthcare Expenditures (USD)**	**95%CI**	**Marginal Effects (USD)**	**95%CI**
**Medical healthcare expenditure**				
Treated	1805.6	(1600.8, 2010.4)	Ref.	
Untreated	2875.1	(1600.6, 4149.6)	1,069.5	(-195.2, 2334.3)
**Pharmaceutical healthcare expenditure**				
Treated	813.4	(692.5, 934.3)	Ref.	
Untreated	833.9	(466.5, 1201.4)	20.5	(-345.5, 386.5)
**Dental healthcare expenditure**				
Treated	439.3	(406.4, 472.2)	Ref.	
Untreated	149.2	(19.7, 278.6)	-290.1***	(-430.1, -150.2)
**Overall healthcare expenditure**				
Treated	2484.8	(2179.7, 2790.0)	Ref.	
Untreated	2933.6	(1606.6, 4260.7)	448.8	(-894.7, 1792.3)

***$p < 0.001$

Note: Marginal effects were derived from a two-part model. Estimates adjusted for age, sex, number of remaining teeth, dental visit in the past year, presence of a primary care dentist, smoking status, and CCI score.

Abbreviation: USD, United States dollar; CI, confidence intervals; Ref, reference; CCI, Charlson Comorbidity Index.

(c) Results of two-part model for annual healthcare expenditures.

	First-part		Second-part	
	**OR**	**95%CI**	**Coefficient**	**95%CI**
**Medical healthcare expenditures**				
Treated (Ref. Untreated)	-0.12	-1.56, 1.32	1,163.4	-182.0, 2,508.7
**Pharmaceutical healthcare expenditures**				
Treated (Ref. Untreated)	-0.55	-1.49, 0.38	85.1	-345.9, 516.1
**Dental healthcare expenditures**				
Treated (Ref. Untreated)	-2.64***	-3.65, -1.62	-176.6	-431.2, 78.0
**Overall healthcare expenditures**				
Treated (Ref. Untreated)	-1.52	-3.30, 0.26	639.3	-798.0, 2,076.7

*p < 0.05; **p < 0.01; ***p < 0.001

Note: Odds ratios are presented for the logit model; coefficients are shown for the GLM model.
Abbreviation: OR, Odds ratio; CI, confidence intervals; Ref, reference; GLM, Generalized linear model.
