# Supplementary material for: Impact of leaving periodontal disease untreated on healthcare expenditures: A retrospective cohort study
Source: J Periodontol. 2026 Apr 19;97(9):2003–12. doi: 10.1002/jper.70136 (PMC13568914; doi:10.1002/jper.70136)
Supplement: Supplementary file 5 [file JPER-97-2003-s005.pdf]

Supplementary Table 4. Top five most frequently billed medical service procedures during the follow-up period, by periodontal treatment status (n = 652).

	**Treated**				**Untreated**		
	Procedure name	Procedure code	Frequency (%)		Procedure name	Procedure code	Frequency (%)
1	Revisit fee	112007410	9.0	1	Prescription issuance fee	120002910	13.2
2	Medical billing statement issuance fee	112015770	7.2	2	Medical billing statement issuance fee	112015770	7.6
3	Outpatient management fee	112011010	6.0	3	Initial consultation fee	111000110	5.7
4	Prescription issuance fee	120002910	5.8	4	Revisit fee	112007410	5.7
5	Management fee for specified diseases	113001810	3.3	5	Management fee for specified diseases	113001810	5.7
